# Fingerprint Analysis and Identification of Strains ST309 as a Potential High Risk Clone in a *Pseudomonas aeruginosa* Population Isolated from Children with Bacteremia in Mexico City

**DOI:** 10.3389/fmicb.2017.00313

**Published:** 2017-03-01

**Authors:** Rosario Morales-Espinosa, Gabriela Delgado, Luis F. Espinosa, Dassaev Isselo, José L. Méndez, Cristina Rodriguez, Guadalupe Miranda, Alejandro Cravioto

**Affiliations:** ^1^Departamento de Microbiología y Parasitología, Facultad de Medicina, Universidad Nacional Autónoma de MéxicoMexico City, Mexico; ^2^Servicio de Pediatría, Hospital Regional 36 San Alejandro, IMSSPuebla, Mexico; ^3^Laboratorio de Bacteriología, Facultad de Veterinaria y Zootecnia, Universidad Nacional Autónoma de MéxicoMexico City, Mexico; ^4^Centro Médico Nacional Siglo XXI, Instituto Mexicano del Seguro Social, Unidad de Investigación en Epidemiología HospitalariaMexico City, Mexico; ^5^Global Evaluative Sciences USA, Inc.Seattle, WA, USA

**Keywords:** *Pseudomonas aeruginosa*, *exoS* and *exoU* genes, genomics island, TFP alleles, ST309, bacteremia, children

## Abstract

*Pseudomonas aeruginosa* is an opportunistic pathogen and is associated with nosocomial infections. Its ability to thrive in a broad range of environments is due to a large and diverse genome of which its accessory genome is part. The objective of this study was to characterize *P. aeruginosa* strains isolated from children who developed bacteremia, using pulse-field gel electrophoresis, and in terms of its genomic islands, virulence genes, multilocus sequence type, and antimicrobial susceptibility. Our results showed that *P. aeruginosa* strains presented the seven virulence genes: *toxA, lasB, lecA, algR, plcH, phzA*1, and *toxR*, a type IV pilin alleles (TFP) group I or II. Additionally, we detected a novel pilin and accessory gene, expanding the number of TFP alleles to group VI. All strains presented the PAPI-2 Island and the majority were *exoU*+ and *exoS*+ genotype. Ten percent of the strains were multi-drug resistant phenotype, 18% extensively drug-resistant, 68% moderately resistant and only 3% were susceptible to all the antimicrobial tested. The most prevalent acquired β-Lactamase was KPC. We identified a group of ST309 strains, as a potential high risk clone. Our finding also showed that the strains isolated from patients with bacteremia have important virulence factors involved in colonization and dissemination as: a TFP group I or II; the presence of the *exoU* gene within the PAPI-2 island and the presence of the *exoS* gene.

## Introduction

*Pseudomonas aeruginosa* is a Gram-negative bacterium, which is categorized as an opportunistic pathogen due to its ability to cause infections mainly in immunocompromised patients. It is a ubiquitous microorganism, metabolically versatile, which is able to adapt to many environments (Gilligan, 1995; Lyczak et al., 2000). An important virulent characteristic is the formation of biofilms and its natural multiresistance to a wide range of antibiotics and disinfectants (Drenkard and Ausubel, 2002; Wolska and Szweda, 2009; Poole, 2011; Rybtke et al., 2015). This microorganism has been associated with nosocomial infections and outbreaks in Intensive Care Units (ICU) for adults, children and neonates (Thuong et al., 2003; Agodi et al., 2007; Zhang et al., 2012). It is a microorganism with the capacity to colonize different surfaces and in hospitals this is common in the colonization of humid sources, such as air conditioning units, sink faucets, and medical equipment (automatic ventilators and humidifiers; Agodi et al., 2007; Kerr and Snelling, 2009).

Approximately, a 30% of the general population carries *P. aeruginosa* on their skin and in their mucosa and intestine (Thuong et al., 2003; Agodi et al., 2007). This bacterium is associated with chronic recurrent infections in patients with cystic fibrosis and it represents a high mortality in children with underlying conditions such as hemato-oncology diseases, cardiovascular surgeries, extended hospitalization in the ICU, gastrointestinal malformations, and prematurity (Fergie et al., 1994; Zhang et al., 2012). Some reports have shown that the incidence of bacteremia due to *P. aeruginosa* falls between 0.09 and 3.8 cases per 1,000 patients with a greater frequency in boys (Grisaru-Soen et al., 2000) with underlying conditions, such as hemato-oncological diseases. Nonetheless, *P. aeruginosa* also causes infections, such as ear infections or skin infections in healthy people exposed to poorly chlorinated water in swimming pools or tubs for hydromassage (Mena and Gerba, 2009; Rybtke et al., 2015).

The genome of *P. aeruginosa* is highly variable due to the insertion of different mobile elements, such as genomic and pathogenic islands that contribute to chromosomal organization and genetic content thereby providing versatility to the bacteria that allows for better adaptation to different niches (Shen et al., 2006; Wiehlmann et al., 2007). Horizontal gene transfer (HGT) is a major force in bacterial evolution conferring a great variability between the species (Jolley and Maiden, 2010; Darmon and Leach, 2014).

The majority of the studies related to *P. aeruginosa* and pediatric cohorts have been performed in patients with cystic fibrosis (Cf; Kus et al., 2004; Kidd et al., 2015), which have genetic and phenotypic characteristic well-studied. The distribution of pilin alleles amongst CF human isolates belong to pilin group I. Biofilm production is thought to be a hallmark of chronic colonization of the CF lung; *P. aeruginosa* “hypermutators” can be isolated from 37 to 54% of the patients with chronic CF infections. MutS, a critical component of the mismatch repair system, is commonly lost in hypermutator strains, resulting in elevated mutation rates. *P. aeruginosa* hypermutator strains isolated from chronically infected patients are often more resistant to antibiotics, possess a mucoid phenotype with small-colony variants on culture medium, and lose both the lipopolysaccharide (LPS) O-antigen and motility (Deretic et al., 1994; Mahenthiralingam et al., 1994; Govan and Deretic, 1996; Häußler et al., 1999, 2003; Oliver et al., 2000; Leone et al., 2008; Chung et al., 2012; Kidd et al., 2012; Rybtke et al., 2015). Published data show the importance of *P. aeruginosa* as a cause of bacteremia in patients who develop neutropenia following chemotherapy and the bacterium has been associated with nosocomial infections (Pronovost et al., 2006). However, there is little published data on the genetic characteristics and the susceptibility patterns of *P. aeruginosa* strains isolated from blood samples from children who developed bacteremia and/or neutropenia following chemotherapy (Oliver et al., 2015; Peña et al., 2015). In the present study, we characterized a collection of *P. aeruginosa* strains isolated from the blood of 60 children with a background of underlying conditions that developed bacteremia and neutropenia post-chemotherapy in a highly specialized hospital in Mexico City.

## Materials and methods

### Bacterial stains

A collection of 60 clinical *P. aeruginosa* strains was used in this study. The clinical isolates were isolated from blood sample taken between October 2011 and May 2014. All patients were treated in the Pediatric Hospital at Centro Medico Nacional, Siglo XXI in Mexico City. The project was approved by the Ethics Committee (No. R-2014-3603-44) of the Pediatric Hospital at the Centro Medico Nacional, Instituto Mexicano del Seguro Social. In all cases, the parents or guardians were informed about the nature of the study and were asked to sign a consent form.

The following reference strains were used as positive controls: *P*. *aeruginosa* PA14 strain, which is an isolate from burn (Berkeley, California, USA) (Lee et al., 2006); two strains from *P. aeruginosa* clone C: C strain, is a typical CF isolate (Hannover Medical School, Germany) and SG17M strain, an environmental isolate from a river water in the city of Mulheim, Germany (Römling et al., 1994, 1997; Lee et al., 2014); *P. aeruginosa* PAO1 strain, which is an isolate from wound (Melbourne, Australia) (Holloway, 1955; Lee et al., 2006). All the strains were maintained in 15% glycerol at −70°C. Each strain was biochemically typed using conventional biochemical tests (Murray et al., 1995; Mac Faddin, 2000) and the API20 NE system (Identification system for non-enteric Gram-negative rods. bioMérieux, Inc.).

### Virulence genes and type III secretion system genotype (TTSS) detection

**Figure 1:**
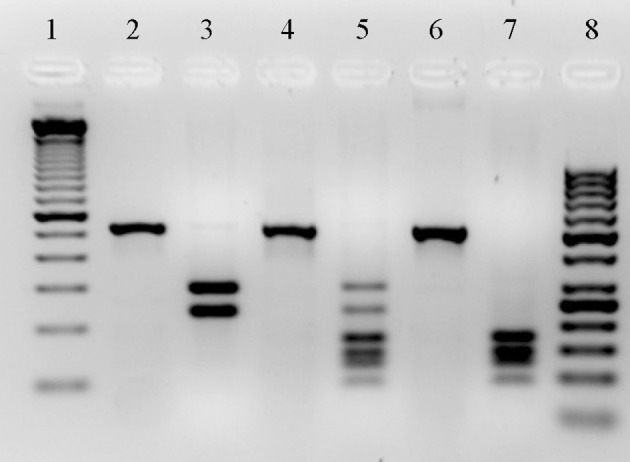
**Restriction Patterns created with *Hinf*I enzyme from PCR products of *exoT* and *exoS* genes**. DNA ladder of 100 bp (Invitrogen by Thermo Fisher Scientific, Inc.) and 50 bp (Thermo Fisher Scientific, Inc.) (lines 1 and 8, respectively). PCR products from *P. aeruginosa* PA14 strain, PAO1 strain and a 1208 clinical strain, the primers: forward 5′ ACTCGTGCGTCCCTTCGTG 3′ and reverse 5′ GATACTCTGCTGACCTCGCTCTC 3′ were used (lines 2, 4, and 6, respectively). Restriction pattern from PA14 strain product, which gave a restriction pattern of two bands for the *exoT* gene: one of 292 bp and the other of 233 bp (line 3). Restriction pattern from PAO1 strain product, which present both *exoT* and *exoS* genes, the bands size correspond to 86, 122, 140, 168, 29, and 233 bp (line 5). Restriction pattern from PCR product of one of our strains, this strain only present the *exoS* gene, which produced a pattern of four bands: 86, 122, 140, and 168 bp (line 7).

Chromosomal DNA was isolated from overnight cultures in Luria broth (Invitrogen, Carlsbad, Ca. USA), of each of the 60 clinical *P. aeruginosa* isolates, as well as from the *P. aeruginosa* control strains (PA14, PAO1, C, and SG17M). DNA was purified from bacteria by miniprep (DNeasy Blood & Tissue Kit QIAGEN, Hilden, Germany) according to the manufacturer's instructions. All DNAs were adjusted to 100 ng/μl at 260/280 nm, using a Tecan Genios equipment. Seven virulence genes (*toxA, lasB, lecA, algR, plcH, phzA*1, and *toxR*) from *P. aeruginosa* were selected and amplified by PCR using Taq DNA polymerase recombinant (Invitrogen, Carlsbad, Ca. USA) and specific primers (Morales-Espinosa et al., 2012). The type III secretion system genes (*exoS, exoT*, and *exoU*) were investigated by PCR. The primers: forward 5′ ACTCGTGCGTCCCTTCGTG 3′ and reverse 5′ GATACTCTGCTGACCTCGCTCTC 3′ were used for *exoS* and *exoT* amplification. The conditions for thermal cycling were: an initial denaturation cycle at 94°C for 2 min, followed by 30 cycles at 94°C for 1 min, the annealing temperature was 55°C for 1 min and 72°C for 1 min with a final cycle of 72°C for 2 min. Subsequently, a restriction pattern from the PCR product was created with the *Hinf* I (Promega Life Science, Madison, Wisconsin. USA) enzyme. The PA14 strain was used as a positive control for *exoT*, which gave a two bands restriction pattern: one of 292 bp and the other of 233 bp. The PAO1 strain was used as positive control for both *exoS* and *exoT* genes, which produced six bands: 86, 122, 140, 168, 233 and 292 bp (Figure 1). The *exoU* gene was detected by PCR using the primers previously documented by Morales-Espinosa (Morales-Espinosa et al., 2012). Detection of the type IV pili (TFP) alleles was carried out according to Kus' characterization (Kus et al., 2004), in which *P. aeruginosa* TFP are divided into five phylogenetic groups. The complete characterization of TFP alleles was made by sequencing (Sanger method, Macrogene Metagenome Next Generation Sequencing [NGS] Service. Korea) of the PCR products from two strains (1,207 and 1,242), which give a greater PCR product (Genbank accession number KX096875 and KX096876).

### Frequency and content of genomic islands

PCR protocols to amplify each gene belonging to PAGI-1 (ORF3, ORF18, and ORF42), PAGI-2 (C22 and C105), PAGI-3 (SG8 and SG100), PAGI-4 (CL22), pKLC102 (CP10, CP44, and CP97), PAPI-1 (the island was detected in its circular form or integrated into the chromosome), and PAPI-2 (*xerC*, RS07-RS08, *exoU*) were carried out according to authors' instructions (Pronovost et al., 2006; Qiu et al., 2006; Klockgether et al., 2007; Morales-Espinosa et al., 2012).

### PFGE (pulse-field gel electrophoresis) analysis

Genomic DNA in agarose blocks was prepared using the method previously described by Liu (Liu et al., 1993) with some modifications such as: allowing a bacterial growth of no more than 12 h, subjecting the bacterial package to lysis twice, deproteinizing the DNA-plugs twice and increasing the number of washes (8) of the DNA-plugs with TBE buffer. The *Spe*I (Roche Diagnostic GmbH. Mannheim, Germany) enzyme was used to obtain the chromosomal profiles. *Spe*I fragments were separated by a CHEF-DR II device (Bio-Rad, USA) and electrophoresis was performed on 1.2 % agarose gels and 0.5X TBE (45 mM Tris, 45 mM Boric acid, 1 mM EDTA) buffer at 10°C with pulse time ramped from 5 to 25 s over 19 h and 5.3 V/cm and a second block with pulse time ramped from 5 to 60 s over 17 h and 5.3 V/cm. The sizes of *Spe*I fragments were estimated using *Xba*I (Roche Diagnostic GmbH. Mannheim, Germany) fragments of *Salmonella* Braenderup global standard H9812. The images were digitized by the Gel Logic 112 imaging system (Kodak, NY, USA). The fingerprinting profile in the PFGE gel was analyzed using BioNumerics v.7.1 (Applied Maths, Belgium) software package. After background subtraction and gel normalization, typing of fingerprint profiles was carried out based on banding similarity and dissimilarity, using the Dice similarity coefficient (Dice, 1945) and the Unweighted Pair Group Method with Arithmetic Mean (UPGMA; Day and Edelsbrunner, 1984) according to average linkage clustering methods.

### MLST (multilocus sequence typing) genotype

MLST was created according to the MLST scheme for *P. aeruginosa* (http://pubmlst.org/paeruginosa) with some modification to the annealing temperatures (according to each specific primer set): *acsA* (64°C), *aroE* (62.3°C), *guaA* (64.1°C), *mutL* (65°C), *nuoD* (56.5°C), *ppsA* (64°C), and *trpE* (63.3°C).

### Antimicrobial susceptibility

To assess the isolates antimicrobial susceptibility, the agar dilution method was used according to the criteria of the Clinical and Laboratory Standards Institute, using the recommended media (CLSI, 2016). ATCC 27853 *Pseudomonas aeruginosa*, ATCC 25922 *Escherichia coli*, ATCC 35218 *E. coli*, ATCC 29213 *Staphylococcus aureus*, and ATCC 29212 *Enterococcus faecalis* were used as quality control. Susceptibility was tested for the following antimicrobial: carbenicillin (Invitrogen Inc., CA, USA); ticarcillin (GlaxoSmithKline, Mexico); piperacillin (Sigma-Aldrich Inc. MO, USA); ticarcillin/clavulanic acid (GlaxoSmithKline, Mexico); piperacillin/tazobactam (Sigma-Aldrich Inc. MO, USA); ceftazidime (Laboratorios Salus S. A. de C. V. Mexico); ceftriaxone (Laboratorios Salus S. A. de C. V. Mexico); cefotaxime (Laboratorios Salus S. A. de C. V. Mexico); cefepime (Laboratorios Salus S. A. de C. V. Mexico); imipenem (Ivax Pharmaceuticals Mexico, S. A. de C. V. Mexico); meropenem (AstraZeneca, Mexico); aztreonam (Sigma-Aldrich Inc., MO, USA); amikacin (Laboratorios Pisa S. A. de C. V. Mexico); gentamicin (Sigma-Aldrich Inc. MO, USA); tobramycin (Alcon Laboratorios S. A. de C. V. Mexico); polymyxin B (GlaxoSmithKline, Mexico); ciprofloxacin (Laboratorio Lemery, S. A. de C. V. Mexico); lomefloxacin (Sigma-Aldrich Inc. MO, USA); norfloxacin (Productos Medix S. A de C. V. Mexico); and levofloxacin (Laboratorio Lemery, S. A. de C. V. Mexico).

### Acquired β-lactamases detection

The most common acquired β-lactamases (Kos et al., 2014; Oliver et al., 2015) were searched for using PCR with specific primers for each group (Table S1). The acquired β-lactamases were sequenced (Macrogene Metagenome Next Generation Sequencing [NGS] Service. Korea) in order to determine the allele type. The conditions for thermal cycling for the β-lactamases genes were: an initial denaturation cycle at 94°C for 2 min, followed by 35 cycles at 94°C for 1 min, the annealing temperature was according to each specific primer set (Table S1) for 1 min and 72°C for 1 min with a final cycle of 72°C for 2 min. All PCR products of each gene were visualized on agarose gel.

## Results

**Figure 2:**
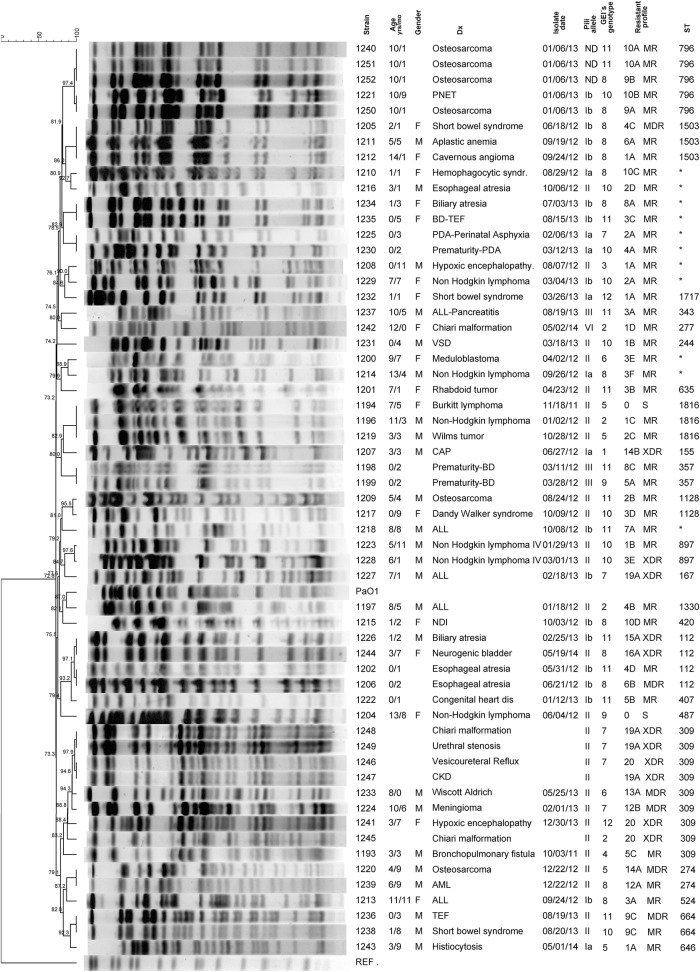
**Pulse-Field gel electrophoresis (PFGE) profile dendrogram and genetic and phenotypic characteristics of *P. aeruginosa* strains isolated from children with bacteremia**. The dendrogram was generated by Dice similarity coefficient (Dice, 1945) and UPGMA (Day and Edelsbrunner, 1984) clustering methods by using PFGE images of *Spe*I digested genomic DNA. The scale bar shows the correlation coefficient (%). Underlying disease (Dx): PNET, primary neuroectodermal tumor; BD-TEF, bronchopulmonary dysplasia-tracheoesophageal fistula; PDA, persistent ductus arteriosus; SGER, severe gastroesophageal reflux; ALL, acute lymphoblastic leukemia; VSD, ventricular septal defect; BD, bronchopulmonary dysplasia; CAP, community acquired pneumonia; NDI, nephrogenic diabetes insipidus; CKD, chronic kidney disease; TEF, tracheoesophageal fistula; AML, acute myeloid leukemia. Asterisk indicate a new ST, which has not been assigned. MDR, Multi-Drug Resistant; MR Moderately Resistant; XDR Extensively drug Resistant. The pili alleles were obtained according to Kus's characterization (Kus et al., 2004), in which *P. aeruginosa* type IV pili are divided into five distinct phylogenetic groups. The GEIs genotype was assigned base on the presence/absence of genomic island, 12 different GEIs genotypes were found (for details see Table S2, Supplementary Material). The resistance profile was formed by a number and a letter: the number indicates how many antibiotics the strain was resistant to; the letters were assigned alphabetically to differentiate among the antimicrobial combinations for which the strains were resistant (detailed information is shown in Table S3, Supplementary Material).

We carried out the characterization of 60 *P. aeruginosa* isolates. All isolates showed biochemical patterns of *P. aeruginosa* (data not shown). The isolates were isolated from children, of whom 65% were female and 35% male. The median age of patients at the time of *P. aeruginosa* bacteremia diagnosis was 5.5 years with a range of 1 month to 14 years and 8 months. All patients admitted to hospital had underlying disease. The most common underlying diseases were hematological and oncological disease (52%) including acute lymphoblastic leukemia, non-Hodgkin lymphoma, solid tumor, hemophagocytic syndrome, histiocytosis and aplastic anemia, and prematurity (24%), other underlying diseases were gastrointestinal malformations, congenital cardiopathy, Wiscott-Aldrich disease, Dandy Walker syndrome, Chiari's malformation, and nephrogenic and diabetes insipidus (Figure 2). Medical records were unavailable for eight patients.

In 28 patients, bacteremia was related to a central catheter. Bacteremia was present in 21 children after chemotherapy treatment, with neutropenia and fever developing. The overall case fatality associated with *P. aeruginosa* bacteremia was 13.3% (8 of 60), who developed septic shock and multi-organ failure (Table 1).

**Table 1:** **Genomic islands detection and susceptibility profile in *P. aeruginosa* strains associated to fatality cases in children with bacteremia**.

**Strain**	**Child gender/age**	**Underlying conditions**	**20 antimicrobials tested**			**GEIs detected**
			**S**	**I**	**R**	
1197	Male/8 years	Acute lymphoblastic leukemia	TI, PI, TM, PT, CA, FE, IM, AZ, AM, GE, TO, PO, CI, LO, NO, LE	CB, CT, ME	CR	PAGI-1, PAPI-1, PAPI-2, pKLC102 [2]
1210	Female/1 year	Hemophagocytic syndrome	IM, ME, AM, GE, TO, PO, CI, LO, NO, LE	CB, TI, TM, CA, FE, AZ	PI, PT, CR, CT	PAPI-1, PAPI-2 [8]
1211	Male/5 years	Aplastic anemia	TI, TM, CA, CR, FE, AZ, AM, GE, TO, PO, CI, LO, NO, LE	CB, PI, CT	PT, IM, ME	PAPI-1, PAPI-2 [8]
1212	Female/14 years	Cavernous angioma	TI, PI, TM, PT, CA, CR, CT, FE, IM, ME, AZ, AM, GE, TO, PO, CI, LO, NO, LE	CB		PAPI-1, PAPI-2 [8]
1220	Male/4 years	Osteosarcoma	AM, GE, TO, PO, NO, LE	CB, TI, PI, TM, CA, FE, AZ, CI, LO	PT, CR, CT, IM, ME	PAPI-1, PAPI-2, pKLC102 [5]
1226	Male/1 year	Biliary atresia	PO, CI, LO, NO, LE	TI	CB, PI, TM, PT, CA, CR, CT, FE, IM, ME, AZ, AM, GE, TO	PAPI-2 [11]
1239	Male/6 years	Acute myeloblastic leukemia	TI, FE, AZ, AM, GE, TO, PO, NO	CB, PI, TM, PT, CA, CI, LO, LE	CR, CT, IM, ME	PAPI-1, PAPI-2 [8]
1241	Female/7 years	Epoxy encephalopathy		PO	CB, TI, PI, TM, PT, CA, CR, CT, FE, IM, ME, AZ, AM, GE, TO, CI, LO, NO, LE	PAGI-1, PAPI-1, PAPI-2 [4]

*S, Susceptible; I, Intermediate resistance; R, Resistant. CB, Carbenicillin; TI, Ticarcillin; PI, Piperacillin; TM, Ticarcillin/Clavulanic acid; PT, Piperacillin/Tazobactam; CA, Ceftazidime; CR, Ceftriaxone; CT, Cefotaxime; FE, Cefepime; IM, Imipenem; ME, Meropenem; AZ, Aztreonam; AM, Amikacin; GE, Gentamicin; TO, Tobramycin; PO, Polymyxin B; CI, Ciprofloxacin; LO, Lomefloxacin; NO, Norfloxacin; LE, Levofloxacin. GEIs, Genomic Islands; [number] correspond to GEIs genotype*.

### Frequency of virulence genes and TTSS genotype detection

All of the isolates isolated from the study's patients presented the seven virulence genes that were amplified by PCR. Due to the high identity between the *exoT* (GenBank accession NC_008463.1) and *exoS* (GenBank accession NC_002516.2) genes (>80%), we could not design specific primers for PCR to amplify each gene. Therefore, we had to use a new strategy that allowed us to differentiate between the detection of *exoS* and *exoT* in each of our strains. The *in silico* analysis of restriction patterns of *exoS* and *exoT* showed that the enzyme *Hinf* I yielded two different patterns between them. Based on this new strategy, 75% of the strains were *exoS*+ and 70% *exoT*+. Of all our strains, 90% presented the *exoU* gene, which was detected by PCR using specific primers. In general, the 67% of our strains were *exoS*+/*exoU*+ genotype, 23% were *exoS*−/*exoU*+ and 10% were *exoS*+/*exoU*−. The *exoS*−/*exoU*− genotype was not found in our study population. Previous studies have reported (Oliver et al., 2015; Peña et al., 2015) that the *exoY* and *exoT* genes are present in all strains. Reason for which, we decided not to detect the *exoY* gene in the present study. However, due to our results where found that the 30% of the isolates are negative *exoT*, now, it does appear necessary to characterize the *exoY* gene in our population and to determine if also there are negative *exoY* strains.

### TFP allele characterization and GEIs detection

With respect to characterization by TFP alleles (Kus et al., 2004), we found that all strains produced a single PCR product ranging in size from ~1.4 to 2.8 kb. Based on PCR product size analysis, 30 out of 60 strains gave a product size of 1,400 bp which was similar to that of group II from the PAO1 reference strain. In addition, three yielded a PCR product of 2,650 bp, as seen for the PA14 control strain and these three strains were determined to belong to group III; 24 strains gave a PCR product (2,800 bp) greater than the PA14 strain; and three strains yielded a PCR product of 1,560 bp. To complete the characterization by TFP allele of the 27 strains with different PCR product sizes, individual PCRs were performed using specific primers for the *tfpO*_*a*_, *tfpO*_*b*_, *tfpY*, and *tfpZ* accessory genes present between *tRNA* and *pilA* (Kus et al., 2004). The results showed that 22 strains amplified the *tfpO* gene (group I), of which seven strains were subgroup Ia and 15 were subgroup Ib, while one strain (1,242 strain) could not be characterized according to Kus's criteria (Kus et al., 2004). Therefore, we selected this strain (1,242) and another strain (1,207) with a 2,800 bp PCR product. Both products were sequenced and analysis of the strain 1,207 showed the presence of the pilin glycosylation gene *tfpO* adjacent to *pilA* (GenBank accession number KX096875), confirming that this strain belongs to group I. However, subgroup 1a or 1b characterization using specific primers for each subgroup (Kus et al., 2004) could not be achieved. Sequence analysis of strain 1,242 showed a novel accessory gene (IS1383), which encodes for a transposase and a new variant of *pilA* gene (GenBank accession number KX096876). The transposase gene has 100% similarity to a transposase gene described in cyclohexylamine-degrading *Pseudomonas plecoglossicida* NyZ12, while the new variant of *pilA* gene presented high identity in its first 345-381 nucleotides with the *pilA* gene of *P. aeruginosa* M1-G, K122-4, and B136-33 strains. According to this result, we identified a new variant of PilA protein and probably, a new TFP allele (Figure S1). We wanted to know if more strains from our study presented the transposase gene and in turn, the new allele. We could not find more strains with this novel TFP allele in our population. We were not been able to sequence the 1,560 bp products of three strains despite three consecutive attempts. However, considering the size of the PCR product, which was very similar to the strain PAO1 Group II, we decided to characterize this *pil* region according to its restriction patterns with *Hph*I enzyme, using the PAO1 strain as reference. The restriction pattern presented for the three strains was the same, with two bands of ~750 and 650 bp (data not shown). However, this pattern was very different to that of the PAO1 strain (675, 425, 147, and 96 bp bands size) and other strains from group II, which suggests greater variability in this region and possibly, the presence of other alleles, as yet not described.

With respect to the detection of genomic islands, we found 12 GEIs genotypes (Table S2), and at least one genomic island was found in all of the strains. The most frequently detected genomic island was PAPI-2 (100%), followed by PAPI-1 (55%), PAGI-1 (47%), and pKLC102 (23%). PAGI-2 was detected in only 3% of the strains; the genomic islands PAGI-3 and PAGI-4 were not detected at all in our study population. The majority of the strains had only two islands; just one strain presented up to five GEIs (PAGI-1, PAGI-2, PAPI-1, PAPI-2, and pKLC102); in six strains four GEIs were detected; in 15 strains 3 GEIs were found and 13 strains had one island only. The genetic content of each GEIs was variable, as has been previously documented (Liang et al., 2001; Klockgether et al., 2007; Morales-Espinosa et al., 2012).

### PFGE and MLST genotype

Using *Spe*I fragment patterns, we found 42 different restriction patterns, of which 29 corresponded to 29 single isolates (unique patterns) and 13 were shared by two or three isolates (Figure 2). Strains 1,195 and 1,203 could not be typed with this method. Although, there were 13 strains that shared chromosomal profiles, the majority of the strains were isolated from unrelated patients, in different hospital services and on different dates. In addition, each strain showed a variable number of GEIs or variability in genetic content and/or a different antimicrobial resistance profile. Only four strains (1,240, 1,250, 1,251, and 1,252) presented the same or similar PFGE patterns and were isolated from same patient in the same day. However, all the isolates had different GEIs number with genetic content and different resistant profile, indicating that this patient had a mixed infection.

The sequence type (ST) in our strains was highly variable, and showed a good correlation with the variability found using the PFGE method. The most frequently detected ST was ST309, which was present in nine strains. These strains were grouped in only one cluster (Figure 2) and all of them shared the TFP group II, six of out nine strains were isolated of urine from patients with urinary tract infection as primary infection, six had the highest resistance profile to around 20 antimicrobials, and four shared the same GEIs genotype. Additionally, there were six strains from this group that presented up to three different β-lactamases (GES20, OXA2, and KPC). A further 21 strains shared STs: as ST796 (5 strains), ST112 (4 strains), ST1503, and ST1816 (3 strains for each), ST357, ST897, and ST664 (2 strains for each), while the remaining 30 strains presented a unique sequence type (Figure 2).

### Antimicrobial susceptibility profile

With respect to susceptibility, only two strains were susceptible to all 20 antimicrobials tested. The 92% of the strains were susceptible to polymyxin B, and between 70 and 85% were sensitive to quinolones, aminoglycosides, cefepime and ceftazidime, while, from 45 to 67% were susceptible to β-lactam antibiotics (Table S3). With respect to resistance, seven strains were resistant to almost all the antimicrobials. The highest rate of intermediate resistance and resistance was observed for carbenicillin (73%) and ceftriaxone (75%). In general, 31.6% (21) of the strains showed resistance to more than 10 antibiotics; 21.6% (13) of strains were multi-resistant more than five antibiotics. We found 43 profiles (phenotypes) of resistance, based on antimicrobials combination for which they were resistant (Table S3).

Regarding adquired β-lactamases detection, 46.6% strains had at least one β-lactamase, with the most frequent detected being KPC (23 strains), followed by OXA-2 (13), GES-20, and GES-5 (9), while VIM-46 was detected in only one strain. Eleven strains presented 2 or 3 β-lactamases, and the greatest concentrated and highest number of adquired β-lactamase types detected was in the ST309 strains.

Based on phenotype stratification for antimicrobial susceptibility or resistance profiles (Magiorakos et al., 2012), 10% were multi-drug resistant (MDR), 18% extensively drug-resistant (XDR), 68% moderately resistant and only 3% were considered susceptible (Figure 2).

### Genetic and phenotypic characteristics of *P. aeruginosa* strains associated to case fatality

Total case fatality associated with *P. aeruginosa* bacteremia was 13.3% (8/60). All the children who died were above 1 year of age and the majority of these patients were diagnosed with a hemato-oncological disease (Table 1). All the children developed septic shock and multi-organ failure. Characterization of the strains showed genetic and phenotypic variability, with four strains sharing chromosomal patterns (1,211, 1,212, 1,220, and 1,239) between them (Table 1 and Figure 2).

## Discussion

European epidemiological surveillance programs show that *P. aeruginosa* is one of the most frequently isolated Gram-negative microorganisms from patients admitted to ICU (Pujol and Limón, 2013). The most important risk factors leading to development of nosocomial infections associated with *P. aeruginosa* in patients are a long period of hospitalization, the existence of a serious pre-existing condition and exposure to invasive procedures (Fergie et al., 1994; Yetkin et al., 2006; Yang et al., 2011). *P. aeruginosa*-associated infections have a high mortality rate due to the presence of virulence factors in the bacterium, innate and acquired multidrug resistance, and immune impairment of the host (Fergie et al., 1994; Lyczak et al., 2000; Corona-Nakamura et al., 2001; Thuong et al., 2003; Poole, 2011). Different studies have shown that *P. aeruginosa* is generally acquired from the hospital environment, person-to-person contact, indirect transmission via contaminated hands, through contaminated respiratory care equipment, catheters, irrigating solutions, and from the use diluted antiseptics and cleaning solutions (Corona-Nakamura et al., 2001; Thuong et al., 2003; Yetkin et al., 2006). Generally, *P. aeruginosa* outbreaks in hospitals are associated with clonally-related strains and through cross transmission in immunocompromised patients with underlying diseases, such as those with malignancies, burns, and prematurity (Agodi et al., 2007; Zhang et al., 2012; Cies et al., 2015). Although *P. aeruginosa* is considered an opportunistic pathogen, it has several virulence factors. These are encoded on plasmids or chromosomal genes, such as *lasB* (encoding for elastase), *toxA* (exotoxin-A), *pilA* (type fimbrial precursor type IV pilin), *plcH* (hemolytic phospholipase C precursor), *phzA1* (phenazine biosynthesis protein), *toxR* (positive transcriptional regulator of *toxA* transcription), *lecA* (lectin; Wick et al., 1990; Walker et al., 1995; Rumbaugh et al., 1999; Woods, 2004; Shen et al., 2006; Wolska and Szweda, 2009; Morita et al., 2015), and four type III effectors: ExoU (phospholipase A2), ExoY (adenylate cyclase), ExoS (ADP-ribosylates numerous proteins, including members of the Ras protein family) and ExoT (a type III cytotoxin that functions as an anti-internalization factor with an N-terminal RhoGAP domain and a C-terminal ADP-ribosyltransferase domain; Sun and Barbieri, 2003; Jia et al., 2006; Cisz et al., 2008; Sun et al., 2012). These last two effectors are closely related to each other and participate in inhibiting phagocytic cells (neutrophil and macrophage function) and in bacterial uptake by epithelial cells (Engel and Balachandran, 2009). The characterization of strains from our current study showed the presence of all virulence genes in 100% of the strains, indicating that these genes are present in the structural integrity of the bacterial chromosome. Contrary to report by other authors (Feltman et al., 2001; Engel and Balachandran, 2009; Oliver et al., 2015; Peña et al., 2015), almost all our strains presented the *exoU* gene. Determination of the TTSS genotype showed that a high percentage was *exoS*+/*exoU*+ genotype. However, not all strains present the *exoT* gene. The presence of both *exoS* and *exoU* genes has been associated with acute infection in humans, such as bacteremia, and correlates with a worse outcome in clinical infections, a higher bacterial burden and a greater risk of death in mechanically ventilated patients (Feltman et al., 2001; Engel and Balachandran, 2009; Peña et al., 2015). While it is true that the results of the present study confirm this important relationship, between the *exoS*+/*exoU*+ genotype and bacteremia, the infections were resolved via antimicrobial treatment, probably due to low resistance of most of our strains to fluoroquinolones, aminoglycosides and ceftazidime, a third generation cephalosporin indicated for the treatment of patients who develop fever associated with neutropenia. On the other hand, registered mortalities in our study were reduced. Analysis of the results showed that there was no association between the *exoS*+/*exoU*+ genotype and risk of death.

The characterization of TFP alleles in our strains show diversity in TFP alleles to a greater extent than previously documented (Kus et al., 2004). A novel TFP allele was detected on the transposase accessory gene located between *tRNA* and *pilA*, which has been documented in other *Pseudomonas* species. A novel group of the *pilA*_VI_ gene with low sequence identity in its 3′ end to other *pilA* groups of different *P. aeruginosa* strains was also detected. The analysis of PilA_VI_ amino acid sequence showed a homology between 31.4 and 42.6% with respect to other groups of PilA (Figure S1). Although, we did not have experimental evidence of the PilA expression, the amino acids sequence is highly homologous in its first 127 aa to other sequences of PilA available in the databases, as it is showed in Figure S1. Using the Swiss-model (homology modeling) software, we obtained a virtual protein structure 100% homologous to *P. aeruginosa* fimbrial protein in its first 125 amino acids (image not shown), although from 126 to 142 aa, no homology was found with respect to the C-terminal region of the same PilA protein. Interestingly, we observed the lack of the two-cysteine residues within of its disulfide-bonded loop (DSL) region, which are involved in the disulfide bond formation contributing to the pilin assembly into fibers and its adhesive capacity (Harvey et al., 2009). The detection of this novel allele supports the notion of horizontal transfer of genes and recombination within homologous regions between bacteria and the incorporation of novel DNA into one of the hypervariable regions of the *P. aeruginosa* chromosome. The singularity of the accessory gene (transposase) and *pilA*_VI_ gene confirmed Kus's observations that each pilin type is stringently associated with a specific accessory gene. Additionally, the presence of the transposase gene immediately adjacent to the tRNA^thr^ gene confirms that a mechanism of bacteriophage-mediated transduction was involved in the generation of the new TFP allele. This is not surprising, since it is known that tRNA genes are hotspots for bacteriophage integration, where we detected greater genetic variability, as seen in the lack of characterization of three of our strains. The characterization of our strains isolated from the blood of children with bacteremia showed the predominance of two TFP alleles (group I and II). In her study, Kus reported a similar percentage of group I pilins within environmental strains and pediatric CF isolates, while in other human isolates, there appears to be an approximately equal distribution of strains within pilin groups I, II, and III (Kus et al., 2004). The characterization of different populations of *P. aeruginosa* isolated from different sources is required in order to determine if there is a correlation between the *pilA* allele and a specificity niche.

In addition to virulence genes, the bacterium acquired foreign DNA in combinations of specific blocks of genes that contributed to virulence and/or adaptation to specific niches. These strain-specific segments of the genome are found in limited chromosomal locations, referred to as genomic islands (GEIs), which are acquired by HGT (Ou et al., 2006; Boyd et al., 2008; Juhas et al., 2009). Depending on their functions, they encode for pathogenicity, symbiosis, fitness, metabolic, or resistance traits (Hacker and Kaper, 2000; Dobrindt et al., 2004; Juhas et al., 2009). A large number of GEIs in the *P. aeruginosa* chromosome have been described, but these GEIs are found in varying numbers in some strains and not in others (Schmidt et al., 1996; Liang et al., 2001; Larbig et al., 2002). In the present study, all the strains isolated from children diagnosed with bacteremia possessed the PAPI-2 Island and more than half of them had PAPI-1 with both islands presenting a mosaic structure. Most of the PAPI-2 genes are related to mobility functions, including integrase genes, transposase genes, one pseudogene, and portions of insertion sequences in addition to the presence of seven ORFs that correspond to hypothetical proteins of unknown function (He et al., 2004). Interestingly, in the right end of PAPI-2 there are two genes that correspond to the *exoU* gene and its chaperone *spcU* (He et al., 2004). As mentioned previously, *exoU* encodes a type III effector (ExoU) that plays an important role in pathogenesis. ExoU is a potent cytotoxin with phospholipase A2 activity, which has been associated with the development of septic shock in an animal model (Kurahashi et al., 1999). The presence of *exoU* in almost all our strains isolated from blood samples taken from patients with bacteremia corroborates the data previously reported by Kurahashi. The presence of *exoU* on PAPI-2 defines this island as a pathogenicity island and it is very likely that the expression of ExoU for *P. aeruginosa* strains facilitates the spread through tissues favoring the arrival of bacteria to bloodstream (He et al., 2004; Kulasekara et al., 2006). On the other hand, PAPI-1 genes are involved in adhesion and/or motility, although, the majority of their genes encode for hypothetical proteins, making this island unique (He et al., 2004; Qiu et al., 2006; Carter et al., 2010; Harrison et al., 2010). This island contains two pairs of two-component regulatory systems, which through mutational analysis have been shown to affect plant and mammalian pathogenesis (He et al., 2004). In addition to all genes being involved in type 4 fimbrial assembly and function in the *pil* chromosomal region, the PAPI-I Island has a set of genes (*pilL, pilN, pilO, pilQ, pilR, pilS, pilT, pilV*, and *pilM*) involved in type IVb pilus biogenesis that contributes to adherence onto synthetic surfaces, such as catheters (Giltner et al., 2011), which provide an entrance to the circulatory system.

The presence of both PAPI-I and PAPI-2 islands in more than half of our strains show that these islands are contributing to *P. aeruginosa* virulence, promoting colonization on catheter surfaces and skin injury with the induction of proinflammatory mediators, and passing the bacteria into the blood system benefitting the survival and fitness of the bacterium. The antimicrobial resistance profiles showed that more than half of our strains isolated from children had a moderate resistance profile, which may provide some explanation as to the low mortality rate reported in our study. The number of MDR and XDR strains detected is still low in the present study (Pediatric Hospital), nevertheless, continuous epidemiological surveillance is necessary to monitor MDR and XDR strain presence considering the continuous admission of patients to different hospital services and horizontal genes transfer from hospital microbiota to patient's native microbiota. Analysis of chromosomal profiles and MLST of the strains showed great genetic variability among our population, indicating that there is no clonal relationship. However, it is striking that in a cluster of nine strains, six were isolated from urine and all nine share the ST309, TFP allele (group II), and have the XDR (six strains) or MDR (two strains) phenotype. There are reports of *P. aeruginosa* high-risk clones circulating in hospitals worldwide, which present specific genetic characteristics (ST111, ST235, and ST175) linked to MDR or XDR phenotype (Cabot et al., 2012; Mulet et al., 2013; Witney et al., 2014; Oliver et al., 2015; Peña et al., 2015). The increasing prevalence of these clones complicates the clinical landscape, limiting therapeutic options and having significant impact on morbidity and mortality. The presence in our population of strains ST309 linked to the MDR or XDR phenotype, make them a potential high-risk clone, which was not documented as such, previously. However, it is important to highlight that in our study, this clone was not associated with any of the mortality cases. Studies in other populations and hospitals settings are recommended in order to determine the presence of ST309 as part of a potential high risk clone, its distribution throughout hospitals in Mexico and its importance to the severity of the clinical outcome.

Based on the overall analysis of the results obtained in this study, we found that the strains of *P. aeruginosa* causing bacteremia in each child harbored *exoU* and *exoS*. This results are support for the Berthelot's observations (Berthelot et al., 2003), who characterized genetically and phenotypically 92 *P. aeruginosa* strains isolated from blood: where, they identified four groups of strains (TTSS types) according to level of type III protein secretion and kinetics of cytotoxicity. Additionally, they made the detection of *exoU* and *exoS* genes by real-time PCR. They found a strong correlation among *exoU*+ and *exoS*+ genotype and TTSS phenotype. They concluded that the most of the bacteremic strains (80%) were strongly cytotoxic for macrophages and that the ExoU-secreting isolates killed the phagocytes more rapidly. Based on Berthelot's study, we can deduce that our strains being cytotoxic. It is likely that *exoU* was acquired through horizontal transfer of PAPI-2 from one strains to other. It also appears likely that the patients were carrying the majority of the strains prior to hospital admission and immunosuppression caused by underlying disease favored the multiplication of microorganisms and adherence to catheter surfaces. The presence of *exoU* on PAPI-2 island gives bacteria the ability to disseminate into the circulation and produce bacteremia, and in some cases the development of septic shock (Engel and Balachandran, 2009).

We identified a reduce number of exogenous β-lactamases among strains, with KPC β-lactamase being the most frequent. However, the presence of a potential high-risk clone, ST309 with a MDR or XDR phenotype, circulating throughout of our hospital could create a serious health problem.

Multiresistant bacteria serve as hosts for the multiple genetic elements (genes, integrons, transposons, and plasmids) that confer their antibiotic resistance phenotypes. This important characteristic allows to the bacteria to be a “successful” bacterial strain, which is an extremely effective vehicle for the dissemination of any genetic element (s) for at least two reasons: (a) all of the hosted resistance elements are transmitted vertically (i.e., from mother to daughter cells) by virtue of the strain's spread and its increasing prevalence and (b) a successful strain has multiple opportunities to act as a donor and to transfer its resistance elements horizontally to other strains, species or genera (Maatallah et al., 2011; Woodford et al., 2011). So that, the identification of a successful multiresistant strain or clone should receive prompt attention to avoid HGT of antimicrobial resistance into bacterial populations, and its dissemination to different hospitals and different regions.

Additionally a high-risk clone should have important characteristics: (a) to be pathogenic (to have virulence factors); (b) to have a resistance profile to at least three groups of antibiotics (extensive drug resistance) and (c) to be present in different places (Woodford et al., 2011). ST309 strains have been documented in France, Australia, Malaysia and even Brazil, which have been isolated from water and some clinical samples such as bronchial lavage, blood, and urinary tract (*P. aeruginosa* PubMLST website).

Despite the fact that the relative contributions of endogenous and exogenous sources to *P. aeruginosa* acquisition are not well-established. At this moment, we can assume that in our study hospital, *P. aeruginosa* infections are not the result of epidemic outbreaks, since the strains associated with infection were highly variable and they were not acquired in the hospital setting.

## Conclusions

To conclude, genetic and phenotypic characterization of 60 isolates of *P. aeruginosa* associated with blood infections in children admitted to a highly specialized hospital in Mexico, showed that the infections were caused by strains with great diversity in their accessory genome. In the majority of the cases, there was no cross-infection between patients associated with a single clone. The *P. aeruginosa* strains isolated from blood and involved in bacteremia were TFP allele group I and II, and cytotoxic (*exoU*+ and *exoS*+). The results support the idea that the presence of PAPI-I and PAPI-2 in the strains contributed to greater virulence, which is associated with better adherence and dissemination into the bloodstream leading to an increased risk of septicemia. We identified the presence of ST309 strains isolated from urinary tract, which possess virulence genes, an extensive drug resistance phenotype, important characteristics that would contribute to make them a potential high-risk clone.

## Author contributions

RM, conceived and designed the experiment, wrote the paper. GM and CR critical review of the article and review the article for intellectual content. RM, GD, and AC, Final approval of the version to be published. JM, CR, performed the experiments of Antimicrobial susceptibility and made the analysis of results. GD, made all genetic analysis corresponding to MLST, Type III Secretion System Genotype and Analyzed in general all results. LE and CR, Designed the primers and performed the experiments for the characterization of the β-lactamase genes and MLST and determined TFSS genotype. DI performed the experiments for PFGE, virulence genes and GEIs determination.

## Funding

This research was funded by DGAPA-PAPIIT Grant number IN212513 from Universidad Nacional Autónoma de México. The funder has no role in study design, data collection, and analysis, decision to publish, or preparation of the manuscript.

### Conflict of interest statement

The authors declare that the research was conducted in the absence of any commercial or financial relationships that could be construed as a potential conflict of interest.

## References

[B1] Agodi A. Barchitta M. Cipresso R. Giaquinta L. Romeo M. A. Denaro C. (2007). *Pseudomonas aeruginosa* carriage, colonization, and infection in ICU patients. Intensive Care Med. 33, 1155–1161. 10.1007/s00134-007-0671-6 17503016

[B2] Berthelot P. Attree I. Plésiat P. Chabert J. de Bentzmann S. Pozzetto B.. (2003). Genotypic and phenotypic analysis of type III secretion system in a cohort of *Pseudomonas aeruginosa* bacteremia isolates: evidence for a possible association between O serotypes and *exo* genes. J. Infect. Dis. 188, 512–518. 10.1086/377000 12898437

[B3] Boyd E. F. Almagro-Moreno S. Parent M. A. (2008). Genomic islands are dynamic, ancient integrative elements in bacterial evolution. Trends Microbiol. 17, 47–53. 10.1016/j.tim.2008.11.003 19162481

[B4] Cabot G. Ocampo-Sosa A. A. Domínguez M. A. Gago J. F. Juan C. Tubau F.. (2012). Genetic markers of widespread extensively drug-resistant *Pseudomonas aeruginosa* high-risk clones. Antimicrob. Agents Chemother. 56, 6349–6357. 10.1128/AAC.01388-12 23045355 PMC3497190

[B5] Carter M. Q. Chen J. Lory S. (2010). The *Pseudomonas aeruginosa* pathogenicity island PAPI-1 is transferred via a novel type IV nilus. J. Bacteriol. 192, 3249–3258. 10.1128/JB.00041-10 20363934 PMC2897670

[B6] Chung J. C. S. Becq J. Fraser L. Schulz-Trieglaff O. Bond N. J. Foweraker J.. (2012). Genomic variation among contemporary *Pseudomonas aeruginosa* isolates from chronically infected cystic fibrosis patients. J Bacteriol. 194, 4857–4866. 10.1128/JB.01050-12 22753054 PMC3430303

[B7] Cies J. J. Jain J. Kuti J. L. (2015). Population pharmacokinetics of the piperacillin component of piperacillin/ tazobactam in pediatric oncology patients with fever and neutropenia. Pediatr. Blood Cancer 62, 477–482. 10.1002/pbc.25287 25328131

[B8] Cisz M. Lee P. Rietsch A. (2008). ExoS controls the cell contact-mediated switch to effector secretion in *Pseudomonas aeruginosa*. J. Bacteriol. 190, 2726–2738. 10.1128/JB.01553-07 18039770 PMC2293250

[B9] Clinical Laboratory Standards Institute (CLSI) (2016). Performance Standards for Antimicrobial Susceptibility Testing. Twenty-Sixth Informational Supplement. M100–S26. Wayne, PA: CLSI.

[B10] Corona-Nakamura A. L. Miranda-Novales M. G. Leaños-Miranda B. Portillo-Gómez L. Hernández-Chávez A. Anthor-Rendón J.. (2001). Epidemiologic study of *Pseudomonas aeruginosa* in critical patients and reservoirs. Arch. Med. Res. 32, 238–242. 10.1016/S0188-4409(01)00267-3 11395191

[B11] Darmon E. Leach D. R. F. (2014). Bacterial genome instability. Microbiol. Mol. Biol. Rev. 78, 1–39. 10.1128/MMBR.00035-13 24600039 PMC3957733

[B12] Day W. H. E. Edelsbrunner H. (1984). Efficient algorithms for agglomerative hierarchical clustering methods. J. Classif. 1, 7–24. 10.1007/BF01890115

[B13] Deretic V. Schurr M. J. Boucher J. C. Martin D. W. (1994). Conversion of *Pseudomonas aeruginosa* to mucoidy in cystic fibrosis: environmental stress and regulation of bacterial virulence by alternative sigma factors. J. Bacteriol. 176, 2773–2780. 10.1128/jb.176.10.2773-2780.1994 8188579 PMC205429

[B14] Dice L. R. (1945). Measures of the amount of ecologic association between species. Ecology 26, 297–302. 10.2307/1932409

[B15] Dobrindt U. Hochhut B. Hentschel U. Hacker J. (2004). Genomic islands in pathogenic and environmental microorganisms. Nat. Rev. Microbiol. 2, 414–424. 10.1038/nrmicro884 15100694

[B16] Drenkard E. Ausubel F. M. (2002). *Pseudomonas* biofilm formation and antibiotic resistance are linked to phenotypic variation. Nature 416, 740–743. 10.1038/416740a 11961556

[B17] Engel J. Balachandran P. (2009). Role of *Pseudomonas aeruginosa* type III effectors in disease. Curr. Opin. Microbiol. 12, 61–66. 10.1016/j.mib.2008.12.007 19168385

[B18] Feltman H. Schulert G. Khan S. Jain M. Peterson L. Hauser A. R. (2001). Prevalence of type III secretion genes in clinical and environmental isolates of *Pseudomonas aeruginosa*. Microbiology 147, 2659–2669. 10.1099/00221287-147-10-2659 11577145

[B19] Fergie J. E. Shema S. J. Lott L. Crawford R. P. C. (1994). *Pseudomonas aeruginosa* bacteremia in immunocompromised children: analysis of factors associated with a poor outcome. Clin. Infect. Dis. 18, 390–394. 10.1093/clinids/18.3.390 8011821

[B20] Gilligan P. H. (1995). Pseudomonas, in Manual de Microbiologia Clinica, eds Murray P. R. Baron E. J. Pfaller M. A. Tenover F. C. Yolken R. H.(Washington, DC: American Society of Microbiology), 517–525.

[B21] Giltner C. L. Rana N. Lunardo M. N. Hussain A. Q. Burrows L. L. (2011). Evolutionary and functional diversity of the *Pseudomonas* type IVa pilin island. Environ. Microbiol. 13, 250–264. 10.1111/j.1462-2920.2010.02327.x 20738375

[B22] Govan J. R. W. Deretic V. (1996). Microbial pathogenesis in cystic fibrosis: mucoid *Pseudomonas aeruginosa* and *Burkholderia cepacia*. Microbiol. Rev. 60, 539–574. 8840786 10.1128/mr.60.3.539-574.1996 PMC239456

[B23] Grisaru-Soen G. Lerner-Geva L. Keller N. Berger H. Passwell J. H. Barzilai A. (2000). *Pseudomonas aeruginosa* bacteremia in children: analysis of trends in prevalence, antibiotic resistance and prognostic factors. Pediatr. Infect. Dis. J. 19, 959–963. 10.1097/00006454-200010000-00003 11055596

[B24] Hacker J. Kaper J. B. (2000). Pathogenicity islands and the evolution of microbes. Annu. Rev. Microbiol. 54, 641–679. 10.1146/annurev.micro.54.1.641 11018140

[B25] Harrison E. M. Carter M. E. K. Luck S. Ou H. He X. Deng Z.. (2010). Pathogenicity islands PAPI-1 and PAPI-2 contribute individually and synergistically to the virulence of *Pseudomonas aeruginosa* strain PA14. Infect. Immun. 78, 1437–1446. 10.1128/IAI.00621-09 20123716 PMC2849418

[B26] Harvey H. Habash M. Aidoo F. Burrows L. L. (2009). Single-residue changes in the C-terminal disulfide-bonded loop of the *Pseudomonas aeruginosa* type IV pilin influence pilus assembly and twitching motility. J. Bacteriol. 191, 6513–6524. 10.1128/JB.00943-09 19717595 PMC2795284

[B27] Häußler S. Tümmler B. Weissbrodt H. Rohde M. Steinmetz I. (1999). Small-colony variants of *Pseudomonas aeruginosa* in cystic fibrosis. Clin. Infect. Dis. 29, 621–625. 10.1086/598644 10530458

[B28] Häußler S. Ziegler I. Löttel A. Götz F. V. Rohde M. Wehmhöhner D.. (2003). Highly adherent small-colony variants of *Pseudomonas aeruginosa* in cystic fibrosis lung infection. J. Med. Microbiol. 52, 295–301. 10.1099/jmm.0.05069-0 12676867

[B29] He J. Baldini R. L. Déziel E. Saucier M. Zhang Q. Liberati N. T.. (2004). The broad host range pathogen *Pseudomonas aeruginosa* strain PA14 carries two pathogenicity islands harboring plant and animal virulence genes. Proc. Natl. Acad. Sci. U.S.A. 101, 2530–2535. 10.1073/pnas.0304622101 14983043 PMC356984

[B30] Holloway B. W. (1955). Genetic recombination in *Pseudomonas aeruginosa*. J. Gen. Microbiol. 13, 572–581. 10.1099/00221287-13-3-572 13278508

[B31] Jia J. Wang Y. Zhou L. Jin S. (2006). Expression of *Pseudomonas aeruginosa* toxin ExoS effectively induces apoptosis in Host Cells. Infect. Immun. 74, 6557–6570. 10.1128/IAI.00591-06 16966406 PMC1698105

[B32] Jolley K. A. Maiden M. C. J. (2010). BIGSdb: scalable analysis of bacterial genome variation at the population level. BMC Bioinformatics 11:595. 10.1186/1471-2105-11-595 21143983 PMC3004885

[B33] Juhas M. van der Meer J. R. Gaillard M. Harding R. M. Hood D. W.. (2009). Genomic islands: tools of bacterial horizontal gene transfer and evolution. FEMS Microbiol. Rev. 33, 376–393. 10.1111/j.1574-6976.2008.00136.x 19178566 PMC2704930

[B34] Kerr K. G. Snelling A. M. (2009). *Pseudomonas aeruginosa*: a formidable and ever-present adversary. J. Hosp. Infect. 73, 338–344. 10.1016/j.jhin.2009.04.020 19699552

[B35] Kidd T. J. Magalhães R. J. S. Paynter S. Bell S. C. Grimwood K. Armstrong D. S.. (2015). The social network of cystic fibrosis center care and shared *Pseudomonas aeruginosa* strain infection: A cross-sectional analysis. Lancet Respir. Med. 3, 640–650. 10.1016/S2213-2600(15)00228-3 26208994

[B36] Kidd T. J. Ritchie S. R. Ramsay K. A. Grimwood K. Bell S. C. Rainey P. B. (2012). *Pseudomonas aeruginosa* exhibits frequent recombination, but only a limited association between genotype and ecological setting. PLoS ONE 7:e44199. 10.1371/journal.pone.0044199 22970178 PMC3435406

[B37] Klockgether J. Würdemann D. Reva O. Wiehlmann L. Tümmler B. (2007). Diversity of the abundant pKLC102/PAGI-2 family of genomic islands in *Pseudomonas aeruginosa*. J. Bacteriol. 189, 2443–2459. 10.1128/JB.01688-06 17194795 PMC1899365

[B38] Kos V. N. Déraspe M. McLaughlin R. E. Whiteajer J. D. Roy P. H. Alm R. A.. (2014). The resistome of *Pseudomonas aeruginosa* in relationship to phenotypic susceptibility. Antimicrob. Agents Chemother. 59, 427–436. 10.1128/AAC.03954-14 25367914 PMC4291382

[B39] Kulasekara B. R. Kulasekara H. D. Wolfgang M. C. Stevens L. Frank D. W. Lory S. (2006). Acquisition and evolution of the *exoU* locus in *Pseudomonas aeruginosa*. J. Bacteriol. 188, 4037–4050. 10.1128/JB.02000-05 16707695 PMC1482899

[B40] Kurahashi K. Kajikawa O. Sawa T. Ohara M. Gropper M. A. Frank D. W.. (1999). Pathogenesis of septic shock in *Pseudomonas aeruginosa* pneumonia. J. Clin. Invest. 104, 743–750. 10.1016/S2213-2600(15)00228-3 10491409 PMC408437

[B41] Kus J. V. Tullis E. Cvitkovitch D. G. Burrows L. L. (2004). Significant differences in type IV pilin allele distribution among *Pseudomonas aeruginosa* isolates from cystic fibrosis (CF) versus non-CF patients. Microbiology 150, 1315–1326. 10.1099/mic.0.26822-0 15133094

[B42] Larbig K. D. Christmann A. Johann A. Hartsch T. Merkl R. Fritz H.. (2002). Gene islands integrated into tRNA Gly genes confer genome diversity on a *Pseudomonas aeruginosa* clone gene. J. Bacteriol. 184, 6665–6680. 10.1128/JB.184.23.6665 12426355 PMC135438

[B43] Lee C. Peters V. Melefors O. Römling U. (2014). Draft genome sequence of *Pseudomonas aeruginosa* SG17M, an environmental isolate belonging to clone C, prevalent in patients and aquatic habitats. Genome Announc. 2, 2009–2010. 10.1128/genomeA.00186-14 24652978 PMC3961725

[B44] Lee D. G. Urbach J. M. Wu G. Liberati N. T. Feinbaum R. L. Miyata S.. (2006). Genomic analysis reveals that *Pseudomonas aeruginosa* virulence is combinatorial. Genome Biol. 7:R90. 10.1186/gb-2006-7-10-r90 17038190 PMC1794565

[B45] Leone I. Chirillo M. G. Raso T. Zucca M. Savoia D. (2008). Phenotypic and genotypic characterization of *Pseudomonas aeruginosa* from cystic fibrosis patients. Eur. J. Clin. Microbiol. Infect. Dis. 27, 1093–1099. 10.1007/s10096-008-0551-1 18488256

[B46] Liang X. Pham X. Q. Olson M. V. Lory S. (2001). Identification of a genomic island present in the majority of pathogenic isolates of *Pseudomonas aeruginosa*. J. Bacteriol. 183, 843–853. 10.1128/JB.183.3.843-853.2001 11208781 PMC94950

[B47] Liu S. L. Hessel A. Sanderson K. E. (1993). The *XbaI*-*BlnI*-*CeuI* genomic cleavage map of *Salmonella* Typhimurium LT2 determined by double digestion, end labelling, and pulsed-field gel electrophoresis. J. Bacteriol. 175, 4104–4120. 10.1128/jb.175.13.4104-4120.1993 8320226 PMC204840

[B48] Lyczak J. B. Cannon C. L. Pier G. B. (2000). Establishment of *Pseudomonas aeruginosa* infection: lessons from a versatile opportunist. Microbes Infect. 2, 1051–1060. 10.1016/S1286-4579(00)01259-4 10967285

[B49] Maatallah M. Cheriaa J. Backhrouf A. Iversen A. Grundmann H. Do T.. (2011). Population structure of *Pseudomonas aeruginosa* from five Mediterranean countries: evidence for frequent recombination and epidemic occurrence of CC235. PLoS ONE 6:e25617. 10.1371/journal.pone.0025617 21984923 PMC3184967

[B50] Mac Faddin J. F. (2000). Biochemical Test for Identification of Medical Bacteria. Philadelphia, PA: Lippincott Williams and Wilkins.

[B51] Magiorakos A. P. Srinivasan A. Carey R. B. Carmeli Y. Falagas M. E. Giske C. G.. (2012). Multidrug-resistant, extensively drug-resistant and pandrug-resistant bacteria: an international expert proposal for interim standard definitions for acquired resistance. Clin. Microbiol. Infect. 18, 268–281. 10.1111/j.1469-0691.2011.03570.x 21793988

[B52] Mahenthiralingam E. Campbell M. E. Speert D. P. (1994). Nonmotility and phagocytic resistance of *Pseudomonas aeruginosa* isolates from chronically colonized patients with cystic. Infect. Immun. 62, 596–605. 8300217 10.1128/iai.62.2.596-605.1994 PMC186146

[B53] Mena K. D. Gerba C. P. (2009). Risk assessment of *Pseudomonas aeruginosa* in water. Rev. Environ. Contam. Toxicol. 201, 71–115. 10.1007/978-4419-0032-6_3 19484589

[B54] Morales-Espinosa R. Soberón-Chávez G. Delgado-Sapién G. Sandner-Miranda L. Méndez J. L. González-Valencia G.. (2012). Genetic and phenotypic characterization of a *Pseudomonas aeruginosa* population with high frequency of genomic islands. PLoS ONE 7:e37459. 10.1371/journal.pone.0037459 22662157 PMC3360775

[B55] Morita Y. Tomida J. Kawamura Y. (2015). Efflux-mediated fluoroquinolone resistance in the multidrug-resistant *Pseudomonas aeruginosa* clinical isolate PA7: identification of a novel MexS variant involved in upregulation of the mexEF-oprN multidrug efflux operon. Front. Microbiol. 6:8. 10.3389/fmicb.2015.00008 25653649 PMC4301020

[B56] Mulet X. Cabot G. Ocampo-Sosa A. A. Domínguez M. A. Zamorano L. Juan C.. (2013). Biological markers of *Pseudomonas aeruginosa* epidemic high-risk clones. Antimicrob. Agents Chemother. 57, 5527–5535. 10.1128/AAC.01481-13 23979744 PMC3811276

[B57] Murray P. A. Baron E. J. Pfaller M. A. Tenover F. C. Yolken R. H. (1995). Manual of Clinical Microbiology. Washington, DC: American ASM Press (Society for Microbiology).

[B58] Oliver A. Canton R. Campo P. Baquero F. Blazquez J. Cantón R.. (2000). High frequency of hypermutable *Pseudomonas aeruginosa* in cystic fibrosis lung infection. Science. 288, 1251–1253. 10.1126/science.288.5469.1251 10818002

[B59] Oliver A. Mulet X. López-Causapé C. Juan C. (2015). The increasing threat of *Pseudomonas aeruginosa* high-risk clones. Drug Resist. Update 21–22, 41–59. 10.1016/j.drup.2015.08.002 26304792

[B60] Ou H. Chen L. Lonnen J. Chaudhuri R. R. Thani A. B. Smith R.. (2006). A novel strategy for the identification of genomic islands by comparative analysis of the contents and contexts of tRNA sites in closely related bacteria. Nucleic Acids Res. 34, 1–11. 10.1093/nar/gnj005 16414954 PMC1326021

[B61] Peña C. Cabot G. Gómez-Zorrilla S. Zamorano L. Ocampo-Sosa A. Murillas J.. (2015). Influence of virulence genotype and resistance profile in the mortality of *Pseudomonas aeruginosa* bloodstream infections. Clin. Infect. Dis. 60, 539–548. 10.1093/cid/ciu866 25378459

[B62] Poole K. (2011). *Pseudomonas aeruginosa*: resistance to the max. Front Microbiol. 2:65. 10.3389/fmicb.2011.00065 21747788 PMC3128976

[B63] Pronovost P. Needham D. Berenholtz S. Sinopoli D. Chu H. Cosgrove S.. (2006). An intervention to decrease catheter-related bloodstream infections in the ICU. N. Engl. J. Med. 355, 2725–2735. 10.1056/NEJMoa061115 17192537

[B64] Pujol M. Limón E. (2013). Epidemiología general de las infecciones nosocomiales. Sistemas y programas de vigilancia. Enferm. Infecc. Microbiol. Clin. 31, 108–113. 10.1016/j.eimc.2013.01.001 23357654

[B65] Qiu X. Gurkar A. U. Lory S. (2006). Interstrain transfer of the large pathogenicity island (PAPI-1) of *Pseudomonas aeruginosa*. Proc. Natl. Acad. Sci. U.S.A. 103, 19830–19835. 10.1073/pnas.0606810104 17179047 PMC1750864

[B66] Römling U. Schmidt K. D. Tümmler B. (1997). Large genome rearrangements discovered by the detailed analysis of 21 *Pseudomonas aeruginosa* clone C isolates found in environment and disease habitats. J. Mol. Biol. 271, 386–404. 10.1006/jmbi.1997.1186 9268667

[B67] Römling U. Wingender J. Muller H. Tummler B. (1994). A major *Pseudomonas aeruginosa* clone common to patients and aquatic habitats. Appl. Environ. Microbiol. 60, 1734–1738. 8031075 10.1128/aem.60.6.1734-1738.1994 PMC201555

[B68] Rumbaugh K. P. Hamood A. N. Griswold J. A. (1999). Analysis of *Pseudomonas aeruginosa* clinical isolates for possible variations within the virulence genes exotoxin A and exoenzyme S. J. Surg. Res. 82, 95–105. 10.1006/jsre.1998.5523 10068532

[B69] Rybtke M. Hultqvist L. D. Givskov M. Tolker-Nielsen T. (2015). *Pseudomonas aeruginosa* biofilm infections: community structure, antimicrobial tolerance and immune response. J. Mol. Biol. 427, 3628–3645. 10.1016/jmb.2015.08.016 26319792

[B70] Schmidt K. D. Tümmler B. Römling U. (1996). Comparative genome mapping of *Pseudomonas aeruginosa* PAO with *P. aeruginosa* C, which belongs to a major clone in cystic fibrosis patients and aquatic habitats. J. Bacteriol. 178, 85–93. 10.1128/jb.178.1.85-93.1996 8550447 PMC177624

[B71] Shen K. Sayeed S. Antalis P. Gladitz J. Ahmed A. Dice B.. (2006). Extensive genomic plasticity in *Pseudomonas aeruginosa* revealed by identification and distribution studies of novel genes among clinical isolates. Infect. Immun. 74, 5272–5283. 10.1128/IAI.00546-06 16926421 PMC1594838

[B72] Sun J. Barbieri J. T. (2003). *Pseudomonas aeruginosa* ExoT ADP-ribosylates CT10 regulator of kinase (Crk) proteins. J. Biol. Chem. 278, 32794–32800. 10.1074/jbc.M304290200 12807879

[B73] Sun Y. Karmakar M. Taylor P. R. Pearlman E. (2012). ExoS and ExoT ADP Ribosyltransferase activities mediate *Pseudomonas aeruginosa* keratitis by promoting neutrophil apoptosis and bacterial survival. J. Immunol. 188, 1884–1895. 10.4049/jimmunol.1102148 22250085 PMC3273577

[B74] Thuong M. Arvaniti K. Ruimy R. de la Salmonière P. Scanvic-Hameg A. Lucet J. C.. (2003). Epidemiology of *Pseudomonas aeruginosa* and risk factors for carriage acquisition in an intensive care unit. J. Hosp. Infect. 53, 274–282. 10.1053/jhin.2002.1370 12660124

[B75] Walker S. L. Hiremath L. S. Galloway D. R. (1995). ToxR (RegA) activates *E. coli* RNA polymerase to initiate transcription of *Pseudomonas aeruginosa* ToxA. Gene 154, 15–21. 10.1016/0378-1119(94)00870-X 7867943

[B76] Wick M. J. Frank D. W. Storey D. G. Iglewski B. H. (1990). Structure, function and regulation of *Pseudomonas aeruginosa* exotoxin A. Annu. Rev. Microbiol. 44, 335–363. 10.1146/annurev.mi.44.100190.002003 2123620

[B77] Wiehlmann L. Wagner G. Cramer N. Siebert B. Gudowius P. Morales G.. (2007). Population structure of *Pseudomonas aeruginosa*. Proc. Natl. Acad. Sci. U.S.A. 104, 8101–8106. 10.1073/pnas.0609213104 17468398 PMC1876578

[B78] Witney A. Gould K. Pope C. F. Bolt F. Stoker N. G. Cubbon M. D.. (2014). Genome sequencing and characterization of an extensively drug-resistant sequence type 111 serotype O12 hospital outbreak strain of *Pseudomonas aeruginosa*. Clin. Microbiol. Infect. 20, 609–6018. 10.1111/1469-0691.12528 24422878

[B79] Wolska K. Szweda P. (2009). Genetic features of clinical *Pseudomonas aeruginosa* strains. Pol. J. Microbiol. 58, 255–260. 19899619

[B80] Woodford N. Turton J. F. Livermore D. M. (2011). Multiresistant Gram-negative bacteria: the role of high-risk clones in the dissemination of antibiotic resistance. FEMS Microbial Rev. 35, 736–755. 10.1111/j.1574-6976.2011.00268.x 21303394

[B81] Woods D. E. (2004). Comparative genomic analysis of *Pseudomonas aeruginosa* virulence. Trends Microbiol. 12, 437–439. 10.1016/j.tim.2004.08.003 15381190

[B82] Yang M. A. Lee J. Choi E. H. Lee H. J. (2011). *Pseudomonas aeruginosa* bacteremia in children over ten consecutive years: analysis of clinical characteristics, risk factors of multi-drug resistance and clinical outcomes. J. Korean Med. Sci. 26, 612–618. 10.3346/jkms.2011.26.5.612 21532850 PMC3082111

[B83] Yetkin G. Otlu B. Cicek A. Kuzucu C. Durmaz R. (2006). Clinical, microbiologic, and epidemiologic characteristics of *Pseudomonas aeruginosa* infections in a University Hospital, Malatya, Turkey. Am. J. Infect. Control. 34, 188–192. 10.1016/j.ajic.2005.11.010 16679175

[B84] Zhang Q. Smith J. C. Zhu Q. Guo Z. MacDonald N. E. (2012). A five-year review of *Pseudomonas aeruginosa* bacteremia in children hospitalized at a single center in southern China. Int. J. Infect. Dis. 16, e628–e632. 10.1016/j.ijid.2012.03.014 22709682

