# Supplementary material for: Fingerprint Analysis and Identification of Strains ST309 as a Potential High Risk Clone in a Pseudomonas aeruginosa Population Isolated from Children with Bacteremia in Mexico City
Source: Front Microbiol. 2017 Mar 1;8:313. doi: 10.3389/fmicb.2017.00313 (PMC5331068; doi:10.3389/fmicb.2017.00313)
Supplement: Supplementary file 1 [file Table1.DOCX]

**Table S1**. List of primers used for the exogenous β-lactamases genes characterization.

|  | **Forward primer sequence (5’-3’)** | **Reverse primer sequence (5’-3’)** | **Tm** | **PCR product** | **Reference** |
| --- | --- | --- | --- | --- | --- |
|  |  |  |  |  |  |
| **β-lactamase type** |  |  |  |  |  |
| GES | ATGCGCTTCATTCACGCAC | CTATTTGTCCGTGCTCAGG | 55 °C | 864 bp | Poirel, 1999 |
| OXA-gpo II | AAGTTAATGGCAATCCGAATCT | CAGCGTCCGAGTTGACTG | 50 °C | 826 bp | This study |
| KPC | AACCCGATGTGTGCCCATCCG | GCGGCGGTGGTGGGCCAATAG | 55 °C | 1067 bp | Deepjyoti, 2015 |
| VIM | GATGGTGTTTGGTCGCATA | CGAATGCGCAGCACCAG | 56 °C | 390 bp | Poirel, 2010 |
| CTX-M | CGCTTTGCGATGTGCAG | ACCGCGATATCGTTGGT | 55 °C | 550 bp | Ahmed, 2004 |
| IMP | TTGACACTCCATTTACDG | GATYGAGAATTAAGCCACYCT | 56 °C | 139 bp | Poirel, 2010 |
| OXA-gpo I | CTACTTGAAGTGTTGACGCCTT | TAGCCACCAATGATGCCC | 50.5 °C | 955 bp | This study |
| OXA-gpo III | CGAACCCGGAGCCTCATT | GCCGTCCCGACTTGATTG | 54 °C | 944 bp | This study |
| PER | AATTTGGGCTTAGGGCAGAA | ATGAATGTCATTATAAAAGC | 55 °C | 925bp | Claeys, 2000 |
| PSE | ACCGTATTGAGCCTGATTTA | ATTGAAGCCTGTGTTTGAGC | 55 °C | 321 bp | Samuelsen, 2010 |
| TEM | ATGAGTATTCAACATTTCCG | CTGACAGTTACCAATGCTTA | 55 °C | 867 bp | Grimm, 2004 |
| VEB | CGACTTCCATTTCCCGATGC | GGACTCTGCAACAAATACGC | 55 °C | 643 bp | Poirel, 2001 |
| AER | CGCCCTAGACACCACGAA | TGATATAGATGCCAACCACCAG | 51 °C | 725 bp | This study |
|  |  |  |  |  |  |
