# Supplementary material for: Fingerprint Analysis and Identification of Strains ST309 as a Potential High Risk Clone in a Pseudomonas aeruginosa Population Isolated from Children with Bacteremia in Mexico City
Source: Front Microbiol. 2017 Mar 1;8:313. doi: 10.3389/fmicb.2017.00313 (PMC5331068; doi:10.3389/fmicb.2017.00313)
Supplement: Supplementary file 2 [file Table2.DOCX]

**Table S2.** GEIs genotype of *P. aeruginosa* strains isolated from children with bacteremia.

| PAGI-1 | PAGI-2 | PAGI-3 | PAGI-4 | PAPI-1 | PAPI-2 | pKLC-102 | GEIs genotype |
| --- | --- | --- | --- | --- | --- | --- | --- |
|  |  |  |  |  |  |  |  |
| + | + | - | - | + | + | + | 1 |
| + | - | - | - | + | + | + | 2 |
| + | + | - | - | - | + | + | 3 |
| + | - | - | - | + | + | - | 4 |
| - | - | - | - | + | + | + | 5 |
| + | - | - | - | - | + | + | 6 |
| + | - | - | - | + | + | - | 7 |
| - | - | - | - | + | + | - | 8 |
| - | - | - | - | - | + | + | 9 |
| + | - | - | - | - | + | - | 10 |
| - | - | - | - | - | + | - | 11 |
| + | - | - | - | - | - | - | 12 |
|  |  |  |  |  |  |  |  |

The GEIs genotype number was assigned arbitrarily, according to the presence or absence of genomic islands.
