# Supplementary figures and images for: Fingerprint Analysis and Identification of Strains ST309 as a Potential High Risk Clone in a Pseudomonas aeruginosa Population Isolated from Children with Bacteremia in Mexico City
Source: Front Microbiol. 2017 Mar 1;8:313. doi: 10.3389/fmicb.2017.00313 (PMC5331068; doi:10.3389/fmicb.2017.00313)

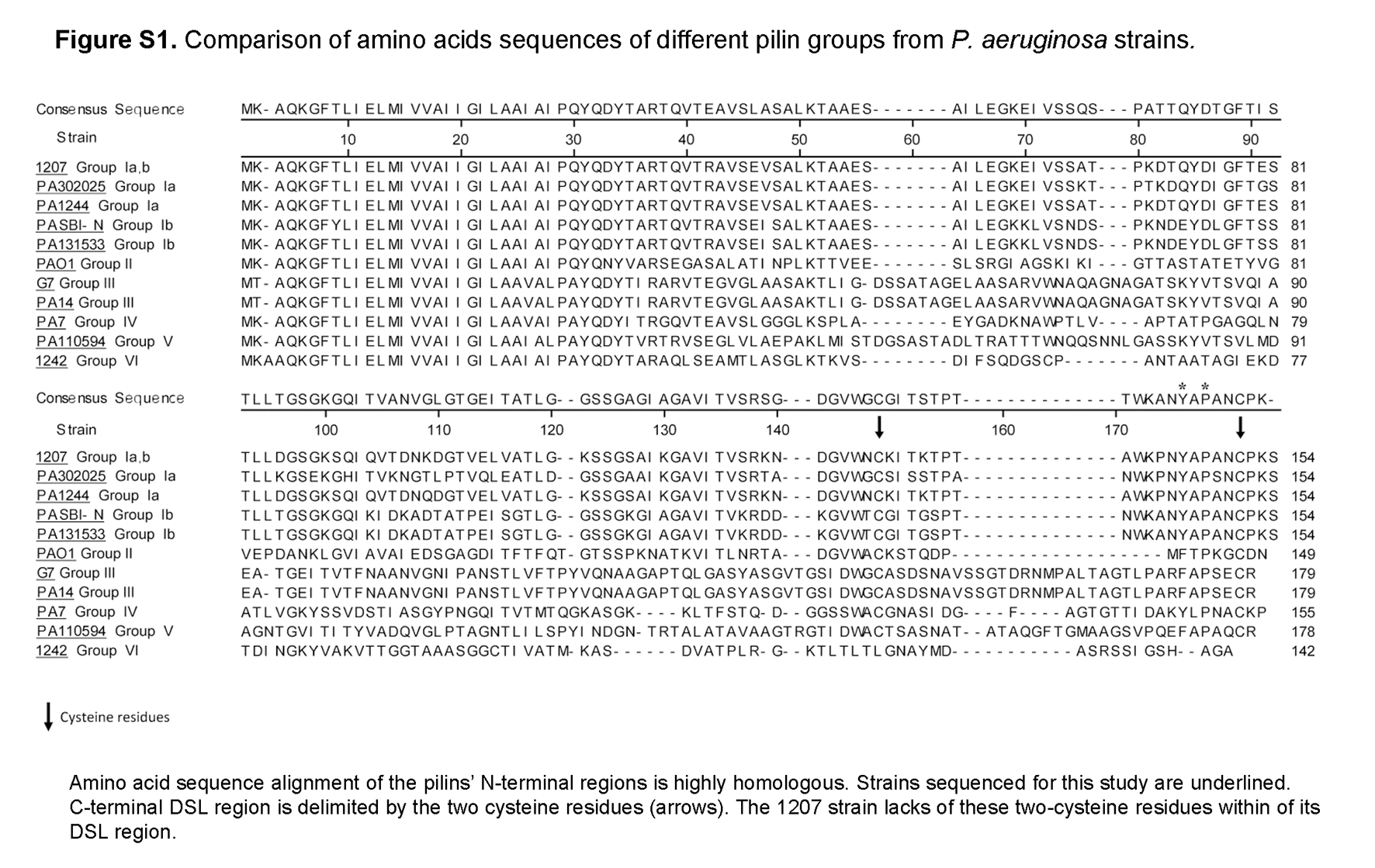

Supplement: Supplementary file 4 [file Image1.TIF]
